# Molecular characterization and application of a novel cytoplasmic male sterility-associated mitochondrial sequence in rice

**DOI:** 10.1186/s12863-015-0205-0

**Published:** 2015-04-30

**Authors:** Yanping Tan, Xin Xu, Chuntai Wang, Gang Cheng, Shaoqing Li, Xuequn Liu

**Affiliations:** Hubei Provincial Key Laboratory for Protection and Application of Special Plants in Wuling Area of China, College of Life Science, South-Central University for Nationalities, Wuhan, 430074 China; Key Laboratory of MOE for Plant Developmental Biology, College of Life Science, Wuhan University, Wuhan, 430072 China

**Keywords:** Chimeric DNA L-sp1, Cytoplasmic male sterility, Mitochondrial genome, Rice

## Abstract

**Background:**

Cytoplasmic male sterility (CMS) is a maternally inherited inability to produce functional pollen found in numerous flowering plant species. CMS is associated with mitochondrial DNA mutation, novel chimeric open reading frames (ORFs), and rearrangement of coding and noncoding regions of the mitochondrial genome.

**Results:**

BLAST (Basic Local Alignment Search Tool) analysis indicated that L-sp1, a new sequence-characterized amplified region, is non-homologous to *atp6*-*orfH79* (or *atp6*-*orf79*) and *WA352* cloned CMS-associated genes. L-sp1 was found in 11 of 102 wild rice accessions belonging to four AA genome species: *Oryza rufipogon*, *Oryza nivara*, *Oryza glumaepatula*, and *Oryza meridionalis*. Using L-sp1, two new CMS lines were developed, from either low natural fertility plants or sterile plants, by backcrossing BC_1_F_1_ with Yuetai B. Northern blot and RT-PCR revealed that L-sp1 was only expressed in the anthers of w1/YTB, w2/YTB, w1/YTB//YTB, and w2/YTB//YTB when in the same cytoplasm background.

**Conclusions:**

L-sp1 is a single-copy chimeric CMS-associated gene found in the mitochondrial genome. It can be expressed in anthers with the same specific cytoplasm background, and will be a useful molecular marker for the development and marker-assisted selection of new CMS lines.

**Electronic supplementary material:**

The online version of this article (doi:10.1186/s12863-015-0205-0) contains supplementary material, which is available to authorized users.

## Background

Cytoplasmic male sterility (CMS) is found in numerous flowering plant species. It is maternally inherited, and causes production of non-functional pollen [1]. In many cases, rearrangement of mitochondrial DNA generates novel chimeric open reading frames (ORFs), resulting in expression of novel polypeptides. Chimeric ORFs are derived from coding and noncoding regions, and are located adjacent to genes coding normal mitochondrial function. The rearrangement of mitochondrial DNA can result in deletion and non-functionality of these mitochondrial genes [2]. Over 50 mitochondrial genes have been identified in various plant species [3-6]. In many species, male fertility can be restored using a nuclear fertility restorer (*Rf*) gene. The CMS/*Rf* system is widely used in the production of hybrid seeds as it eliminates the need for hand emasculation. Moreover, it makes an excellent model system for studying interactions between nuclear and mitochondrial genomes.

Rice (*Oryza sativa* L.) is an important crop, providing a major food source for about half of the world’s population. Since the discovery of the first CMS line [7], over 60 CMS lines, from various origins, have been developed from inter-species, inter-subspecies, and inter-varieties of *Oryza* plants with the AA genome [8]. CMS lines of rice are mainly divided into wild-abortive (WA), Honglian (HL), and Boro II (BT) groups according to distinctive cytological and genetic characteristics [9]. The WA-CMS system was broadly used for hybrid rice production in China by the end of the 20th century. Its irregularly shaped pollen aborts at the uninucleate stage with negative stainability in 1% I_2_-KI solution. Recent studies suggest that the mitochondrial gene, *WA352*, confers WA-CMS by interacting with the nuclear-encoded mitochondrial protein COX11. *WA352*-induced sterility can be suppressed using two restorer-of-fertility (*Rf*) genes [10]. BT-CMS is the most fully characterized CMS system in rice. Its pollen aborts at the trinucleate stage and is partly stainable in 1% I_2_-KI solution, revealing black spherical pollen grains. An unusual chimeric sequence called *orf79* encodes a cytotoxic peptide in the mitochondrial genome of the BT-CMS line [11]. This chimeric sequence includes a small portion of the *cox1* gene, and a sequence of unknown origin [12]. As a new germplasm source, HL-CMS has shown great potential for both hybrid rice production and nucleo-cytoplasmic interaction studies. Its spherically shaped pollen aborts at the dinucleate stage with negative stainability in 1% I_2_-KI solution. Previous studies revealed that coexpression of *atp6-orfH79* might interfere with construction of F_0_F_1_-ATPase during microgenesis [13,14]. Recent studies have focused on the chimeric gene *orfH79* in the HL-CMS line. This chimeric gene shows 97% similarity to *orf79*, and has a 6-base pair (bp) addition at the intercistronic linker between *H-atp6* and *orfH79* that is absent in *orf79*. It also encodes a cytotoxic peptide, and affects the development of male gametophytes and the roots [15,16].

Over 17 *Rf* or loci for different CMS systems have been reported in rice [11,17-24]. With regard to the HL-CMS/*Rf* system, *Rf5* has been finely mapped on chromosome 10 [21]. It encodes a pentatricopeptide repeat protein, and it physically interacts with GRP162, a Gly-rich protein encoding 162 amino acids, to form a restoration of fertility complex that cleaves CMS-associated transcripts and restores fertility [25]. Another *Rf6* gene has been mapped to a region of approximately 200 kb between markers RM3710 and RM22242 on the short arm of chromosome 8 [26]. Presently, three alleles or loci for HL-CMS have been identified in wild rice by genetic and allelic analyses [27]. A synergistic relationship exists between CMS and fertility-restoration-related genes in *Oryza* species [28], and the *Rf* allele interacts with CMS factors in a gene-for-gene manner [29-32]. Thus, other CMS-associated DNA sequences or factors are likely to occur in the HL-CMS/*Rf* system. In this study, we developed a novel sequence-characterized amplified region (SCAR) marker for L-sp1, a chimeric mitochondrial genomic DNA sequence, using random amplification of polymorphic DNA (RAPD) in mitochondrial genomic DNA. Furthermore, L-sp1 can be used for the development of new CMS lines and identification of new cytoplasmic backgrounds through marker-assisted selection (MAS).

## Results

### Development and genetic analysis of SCAR marker

**Figure 1:**
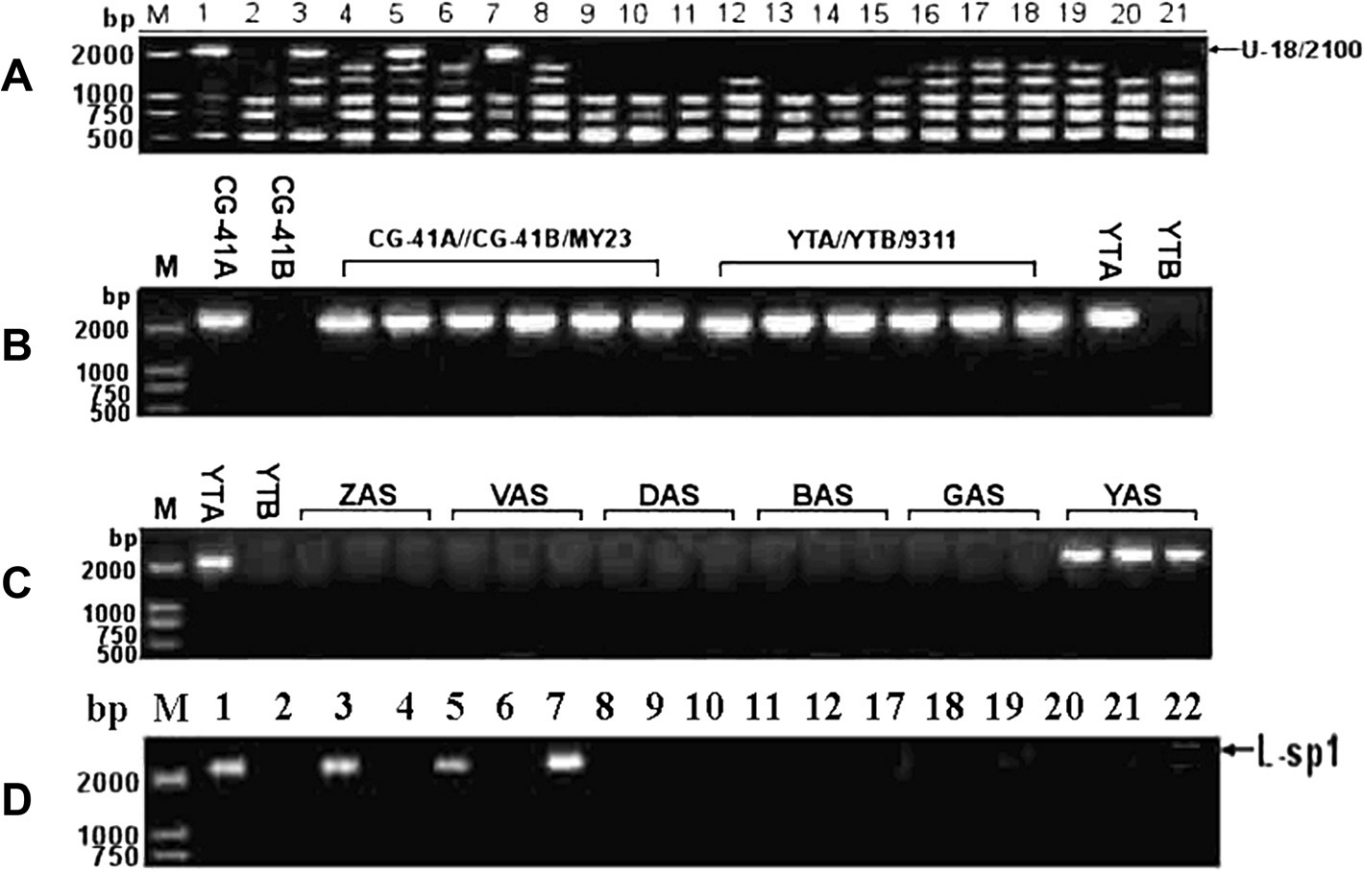
Genetic analysis and verification of L-sp1. **A**: PCR products amplified from genomic DNA of the studied accessions by OPN U-18. **B**: Amplified L-sp1 present in test-cross plants of YTA//YTB/9311 and CG-41A//CG-41B/MY23, and F_2_ plants of HL-2. **C**: Amplified L-sp1 fragment present in plants of different types of CMS lines with the same nucleic background. **D**: Mitochondrial genomic DNA amplified by L-sp1 primers. M: DNA marker DL2000. Numbers above the lanes match to the corresponding plant material No. listed in Table 1.

PCR amplification was performed using total genomic DNA of 28 accessions (Table 1) with 264 random primers (10 nucleotides). A 2100-bp product, named U-18/2100, was amplified when using the RAPD primer OPN U-18 (5′-GAGGTCCACA-3′) with DNA templates from YTA, CG-41A, HL-2, and C-M23 containing HL-type male sterile cytoplasm (Figure 1A). U-18/2100 was recovered, cloned, and sequenced according to TA-cloning protocols. Following U-18/2100 sequence analysis, a SCAR marker was developed and named as L-sp1 (specific primers, H1: 5’-GAGGTCCACATCCTTCAATC-3’; H2: 5’-AGGTCCACAAACCACTGAAG-3’). The genetic nature of the L-sp1 fragment was determined by PCR using total genomic DNA of plants from two different backcross populations: BC_1_F_1_s CG-41A//CG-41B/MY23 and YTA//YTB/9311. These two backgrounds display HL cytoplasmic male sterility and specific nuclear backgrounds (Figure 1B), as did plants from six different types of CMS lines (BC_7_F_1_) with similar SJB nucleic backgrounds (Figure 1C). The L-sp1 fragment was consistently amplified in all plants possessing the HL cytoplasmic male sterility background, and amplification remained constant with changes in the nucleic genome. L-sp1 can be inherited cytoplasmically or maternally, and has specificity for CMS. Based on characteristics of the cytoplasmic genes and the possible relationship between L-sp1 and CMS, we further verified stability and reliability of the SCAR marker using mitochondrial DNA from 18 out of 28 accessions (Table 1) with specific primers H1 and H2. The L-sp1 fragment could be amplified from mitochondrial genomic DNA of YTA, CG-41A, HL-2, and C-M23 (Figure 1D), suggesting that U-18/2100 is related to CMS.

**Table 1:** **The three CMS rice types and F**_**1**_ **offspring used in this study**

**No.**	**Name (Code)**	**Character**	**Type of CMS/** ***Rf*** **system**
1	Yue Tai A, YTA	Sterile line	HL-CMS/*Rf* system
2	Yue Tai B, YTB	Maintainer line	
3	YTA/9311, HL-2	F_1_ hybrid	
4	9311	Restorer line	
5	Cong Guang 41A, CG-41A	Sterile line	
6	Cong Guang 41B, CG-41B	Maintainer line	
7	CG-41A/MY23, C-M23	F_1_ hybrid	
8	MiYang23, MY23	Restorer line	
9	Zhen Shan 97 A, ZSA	Sterile line	WA-CMS/*Rf* system
10	Zhen Shan 97 B, ZSB	Maintainer line	
11	ShanYou 63, SY63	F_1_ hybrid	
12	Ming Hui 63, MH63	Restorer line	
13	Maxie A, MXA	Sterile line	
14	Maxie B, MXB	Maintainer line	
15	Maxie 63, MX63	F_1_ hybrid	
16	Ming Hui 63, MH63	Restorer line	
17	Liu Qian Xin A, QXA	Sterile line	BT-CMS/*Rf* system
18	Liu Qian Xin B, QXB	Maintainer line	
19	HP121, HP121	Restorer line	
20	ShiJin A, SJA	Sterile line	
21	ShiJin B, SJB	Maintainer line	
22	PeiC311, PC311	Restorer line	
23	YTA/SJB, YAS	BC_7_F_1_	Different CMS lines with the same SJB nucleus
24	ZSA/SJB, ZAS	BC_7_F_1_	
25	V20A/SJB, VAS	BC_7_F_1_	
26	DA/SJB, DAS	BC_7_F_1_	
27	BoA/SJB, BAS	BC_7_F_1_	
28	GangA/SJB, GAS	BC_7_F_1_	

Note: ZSA, V20A, DA, BoA, and GangA are different CMS lines developed and applied in China. SJB, Shijing B, a maintainer line from Japonica rice.

### Molecular characterization of mitochondrial SCAR marker

**Figure 2:**
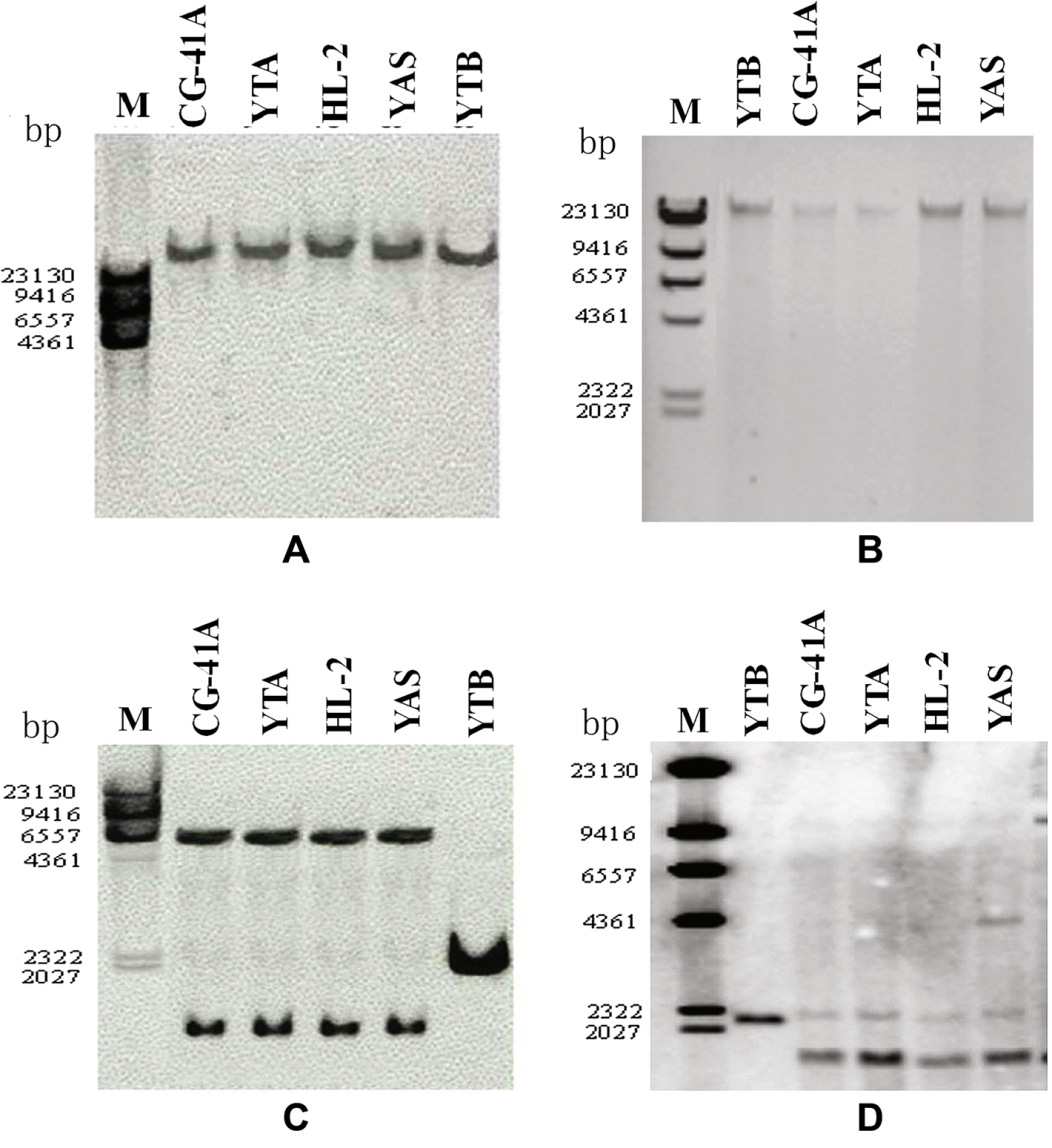
Southern analysis. M: Lambda DNA/*Hind* III Marker. **A**, **B**: Mitochondrial genomic DNA of CG-41A, YTA, HL-2, and YTB, and YAS cut with *Bam*HI. **C**, **D**: Mitochondrial genomic DNA of CG-41A, YTA, HL-2, and YTB, and YAS cut with *Eco*RI. **A**, **C**: Southern analysis with L-sp1 probes. **B**, **D**: Southern analysis with *N-atp6* probes. L-sp1 probe was amplified using the sequence-specific primers H1 and H2, these contain one *Eco*RI restriction site and no *Bam*HI restriction site. *N-atp6* probe was amplified using sequence-specific primers F and R, and also contain one *Eco*RI restriction site and no *Bam*HI restriction site. A part of L-sp1 probe is homologous to a part of the *N-atp6* probe.

To assay HL-CMS specificity and L-sp1 copy number, mitochondrial genomic DNA of CG-41A, YTA, HL-2, YAS, YTB, and of all accessions was digested with *Eco*RI or *Bam*HI and hybridized with L-sp1 probe. A single fragment of 23 kb was found when using L-sp1 (2176 bp specific primer, H1: 5’-GAGGTCCACATCCTTCAATC-3’; H2: 5’-AGGTCCACAAACCACTGAAG-3’) and *N-atp6* (S59890) probes (F: 5’-CAATCCTTGGTAGAGTG-3’; R: 5’-TAATGGCAGTGGGACTCC-3’) for all accessions following digestion with *Bam*HI. The band was the same size in CG-41A, YTA, HL-2, and YAS, but smaller in YTB (Figure 2A and B). Three bands were detected when using the L-sp1 probe following digestion with *Eco*R1, there were two bands detected in CG-41A, YTA, HL-2, and YAS, and one band in YTB (Figure 2C). One band in YTB, and two bands in CG-41A, YTA, HL-2, and YAS were detected when using the *N-atp6* probe following digestion with *Eco*R1 (Figure 2D). These results indicated that L-sp1 was single copy, and could be used as a characteristic molecular marker in the mitochondrial genome.

**Figure 3:**
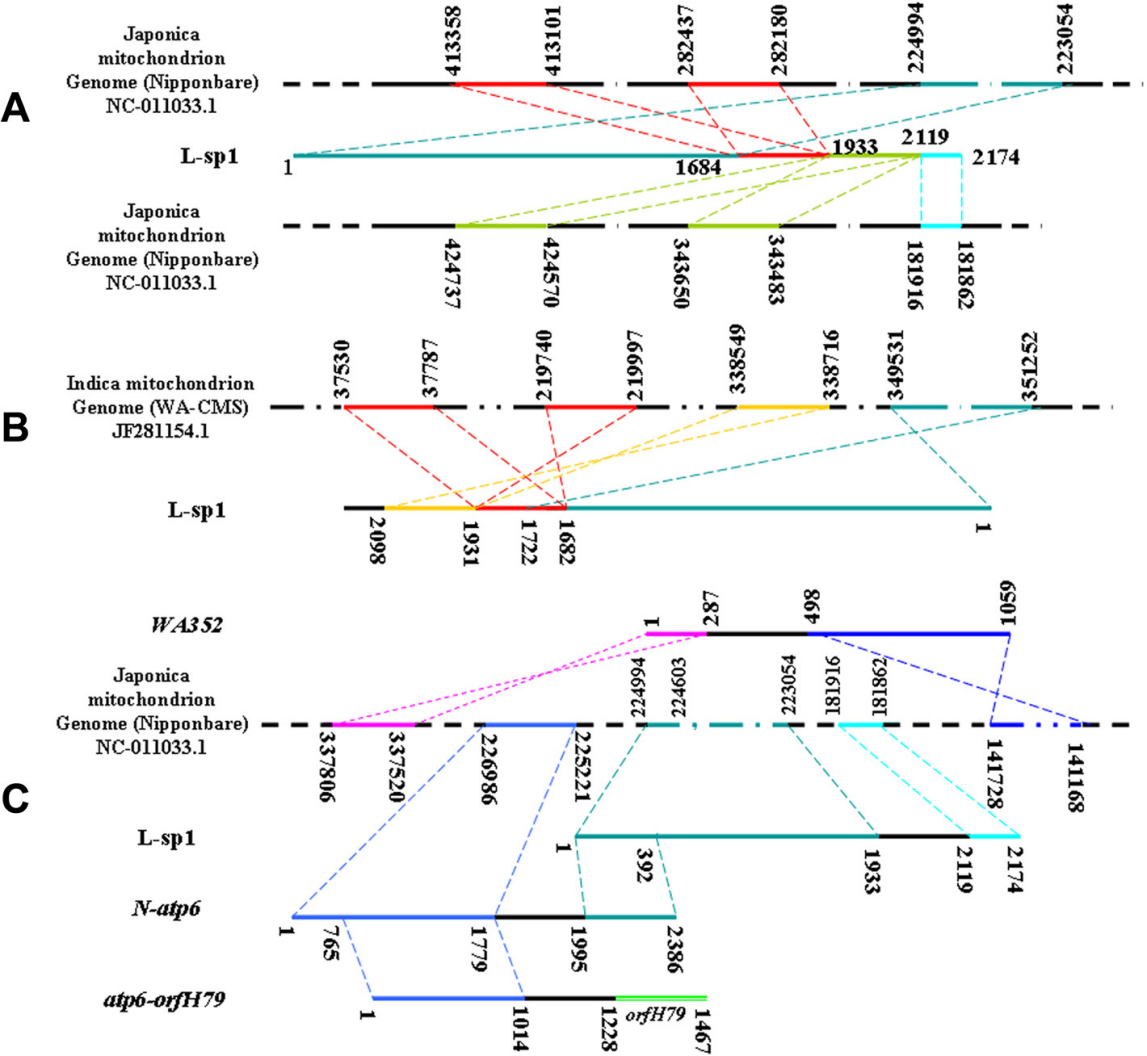
Comparison of sequences among L-sp1, Nipponbare mtDNA, *N-atp6*, and *atp6-orfH79*. **A**: Sequence comparison between L-sp1 and Nipponbare mtDNA. **B**: Sequence comparison between L-sp1 and Indica WA-CMS mtDNA. **C**: Sequence comparison between L-sp1, *N-atp6*, *atp6-orfH79*, and *WA352. Colored lines*: homologous sequence. *Black lines*: non-homologous sequence.

L-sp1 is a chimeric mitochondrial genomic DNA sequence of 2176 bp (HQ267715). When compared with the mitochondrial genomic DNA sequence, L-sp1 was determined to contain four Japonica rice (Nipponbare) DNA fragments (Figure 3A). Except for a 10-bp gap located between bp 1724 and 1725, the sequence from bp 1 to 1933 of L-sp1 showed 99% similarity to bp 224994 to 223054 of the NC_011033.1 clone. The L-sp1 sequence from bp 1684 to 1933 showed 99% similarity to bp 282437 to 282180 and 413358 to 413001 of the same clone. The L-sp1 sequence from bp 1934 to 2100 showed 99% similarity to bp 343650 to 343483 and 424737 to 424570 of the NC_011033.1 clone, and 100% similarity to the cDNA sequence located at 1062 to 1228 bp of the mitochondrial ribosomal protein L5 gene. The remaining 56-bp DNA sequence (bp 2119 to 2174) of L-sp1 was similar to bp 181916 to 181862 of the NC_011033.1 clone.

Sequence comparison of sequences between L-sp1 and indica WA-CMS mitochondrial genomic DNA (Figure 3B) was as follows: the L-sp1 sequence from bp 1 to 1722 showed 98% similarity to bp 349531 to 351252 of JF281154.1; bp 1682 to 1931 showed 97% similarity to bp 37787 to 37530 and 219740 to 219997 of JF281154.1; and bp 1931 to 2098 of L-sp1 showed 100% similarity to bp 338549 to 338716 of JF281154.1. Other sequences shared no homology to the indica WA-CMS mitochondrial genome.

BLAST analysis of L-sp1, *WA352* (AGG40956), *N-atp6*, and *atp6-orfH79* sequences [25] revealed that L-sp1 sequences from bp 1 to 392 were entirely homologous to the 3’ flanking sequence of the ORF of *N-atp6*. Other DNA sequences showed no homology between L-sp1 and *N-atp6*. Furthermore, L-sp1 was non-homologous to the total DNA sequences of both *atp6-orfH79* and *WA352* (Figure 3C).

### Distribution of L-sp1 in the AA genome of wild rice

**Figure 4:**
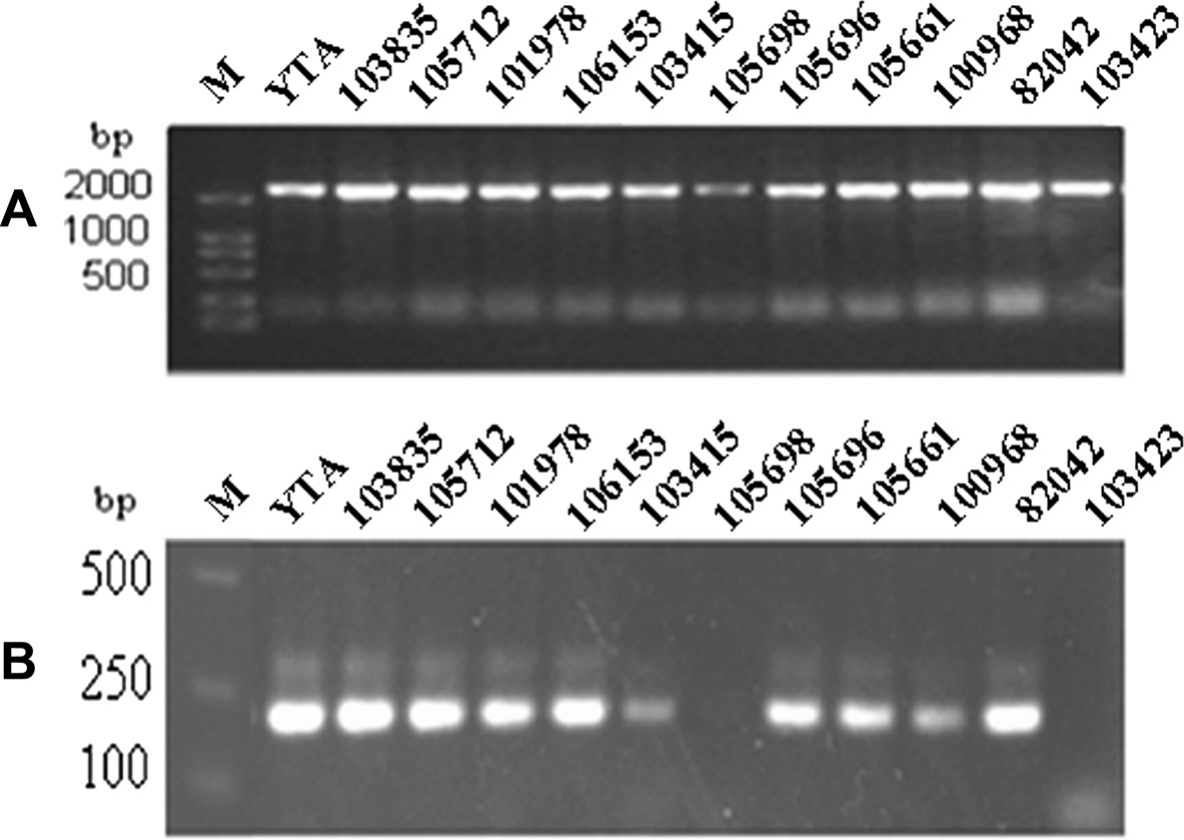
Distribution of L-sp1 in wild rice and determination of cytoplasmic background of HL-type hybrid seeds. **A**: Distribution of L-sp1 in wild rice. **B**: Distribution of *orfH79* (*orf79*) in wild rice. YTA and the 11 strains of wild rice correspond to those listed in Additional file 1: Table S1. M: DNA marker DL2000.

To determine the distribution of L-sp1 in the AA genome of wild rice, PCR amplification was performed using L-sp1 sequence-specific primers H1 and H2. Bands of approximately 2176 bp, the same size as those in YTA, were found in 11 of 102 investigated wild rice accessions (Figure 4A). These 11 accessions belonged to four species: three from *O. rufipogon* (103423, 105696, 105698), five from *O. nivara* (101978, 103415, 103835, 105712, 106153), two from *O. glumaepatula* (100968, 105661), and one from *O. meridionalis* (82042), these accessions came from Cambodia, India, Sri Lanka, Suriname, Brazil, Bangladesh and Laos in Southeast Asia, West Africa, South America, and Oceania, respectively. To analyze the distribution of *orfH79* in the 11 wild rice accessions, PCR was performed using *orfH79* sequence-specific primers (O1: 5′-ATGACAAATCTGCTCCGAT-3′; O2: 5′-TTACTTAGGAAAGACTACAC-3′). This revealed that 9 of the 11 wild accessions amplified the same sized band, approximately 240 bp, as YTA, while the other two accessions (103423, 105698) failed to amplify a PCR product (Figure 4B).

### Development of new CMS lines via backcrosses from accessions containing L-sp1

**Figure 5:**
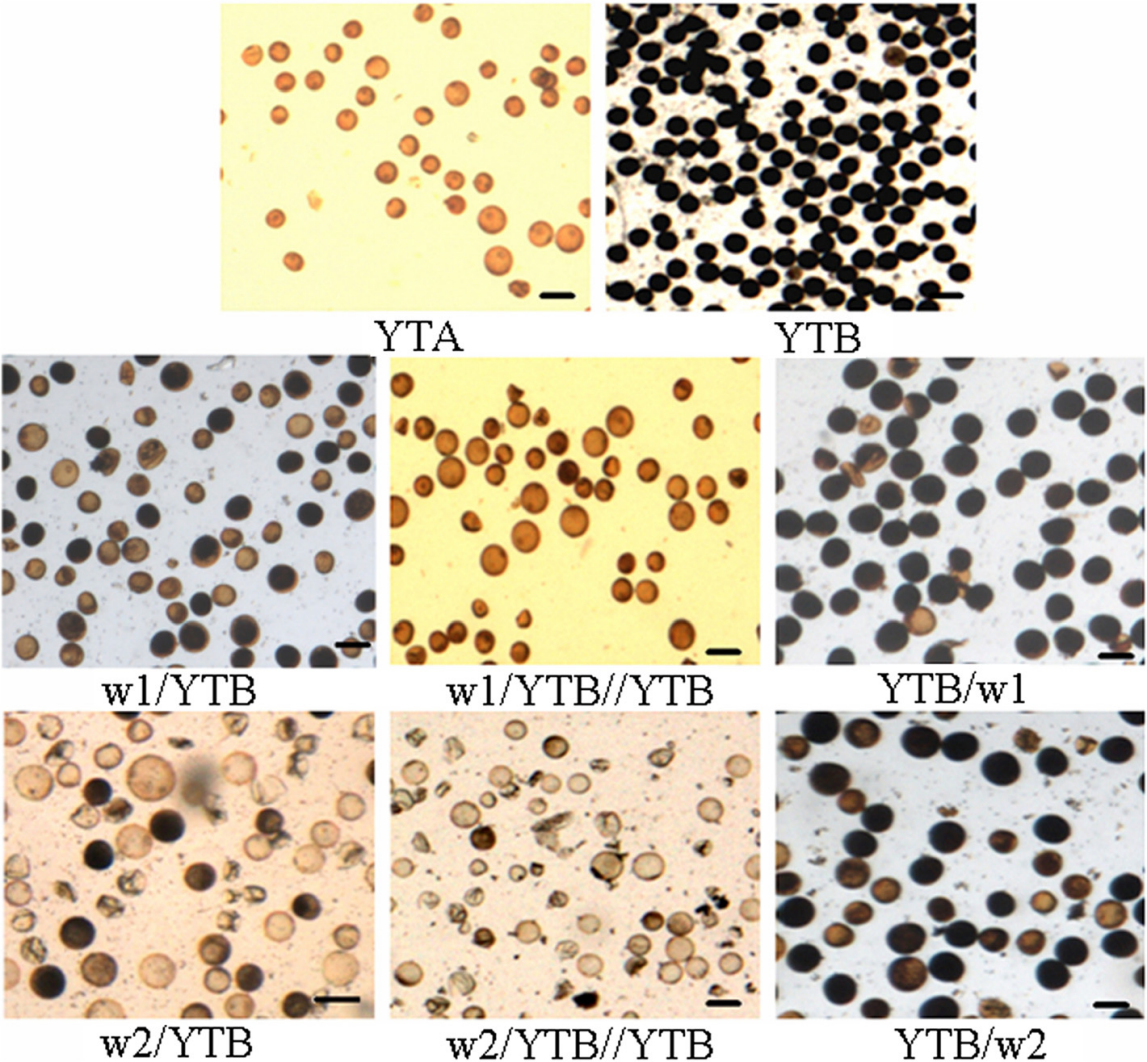
Pollen microspores. Bars = 50 μm.

To validate whether L-sp1 was related to CMS at the molecular genetic level, an interspecies cross was performed using two accessions (103423 and 105698, now named w1 and w2, respectively) carrying L-sp1 as maternal parents with YTB. Fertility analysis revealed the percentage of stainable pollen grains of the F_1_ hybrids w1/YTB and w2/YTB were 10.3% ± 1.5% and 15.8% ± 2.3%, respectively; over 50% of abortive pollen grains were spherical (Figure 5). The seed-setting rates of bagged spikelets for the same crosses were 13.6% ± 1.5% and 23.5% ± 2.5%, respectively (Table 2). To elucidate whether male sterility of the test-cross derived from potential incompatibility between species or subspecies, the HL maintainer YTB was crossed as a female parent with w1 and w2. Fertility assessment revealed that YTB/w1 and YTB/w2 were both fertile (~80% pollen fertility; ~50% seed-setting fertility). This indicated that the fertility of crosses between wild rice and YTB was mainly influenced by the cytoplasm genome as opposed to the nuclear genome in wild rice. Next, fertility of populations derived from BC_1_F_1_ backcrosses of w1/YTB//YTB and w2/YTB//YTB were examined; the spherical abortive grain rate increased from ~25% to ~85%, and fertility of pollen and seed-setting was clearly reduced (Figure 5). Therefore, two new CMS lines could be developed from low fertility and sterile plants belonging to BC_1_F_1_ (w1/YTB//YTB and w2/YTB//YTB) or BCnF_1_ (n, generation number of backcross) maternal parents, by successive backcrossing with YTB.

**Table 2:** **Fertility analysis of hybrid F**_**1**_ **and BC**_**1**_ **F**_**1**_ **backcrossed lines**

**Accessions**	**103423 (w1) %**			**105698 (w2) %**		
Combination	w1/YTB	YTB/w1	w1/YTB//YTB	w2/YTB	YTB/w2	w2/YTB//YTB
Spherical abortive	54.3 ± 2.6		82.5 ± 2.8	61.2 ± 3.4		86.5 ± 2.1
Typical abortive	35.4 ± 1.8		13.0 ± 2.1	23.0 ± 4.5		10.3 ± 2.7
Pollen fertility	10.3 ± 1.5	77.3 ± 3.2	4.5 ± 0.6	15.8 ± 2.3	82.3 ± 1.9	3.2 ± 0.4
Seed-setting fertility	13.6 ± 1.5	45.6 ± 2.4	7.8 ± 1.1	23.5 ± 2.5	54.2 ± 2.2	3.5 ± 2.1

### Genetic and transcript analysis of L-sp1 in new CMS lines

**Figure 6:**
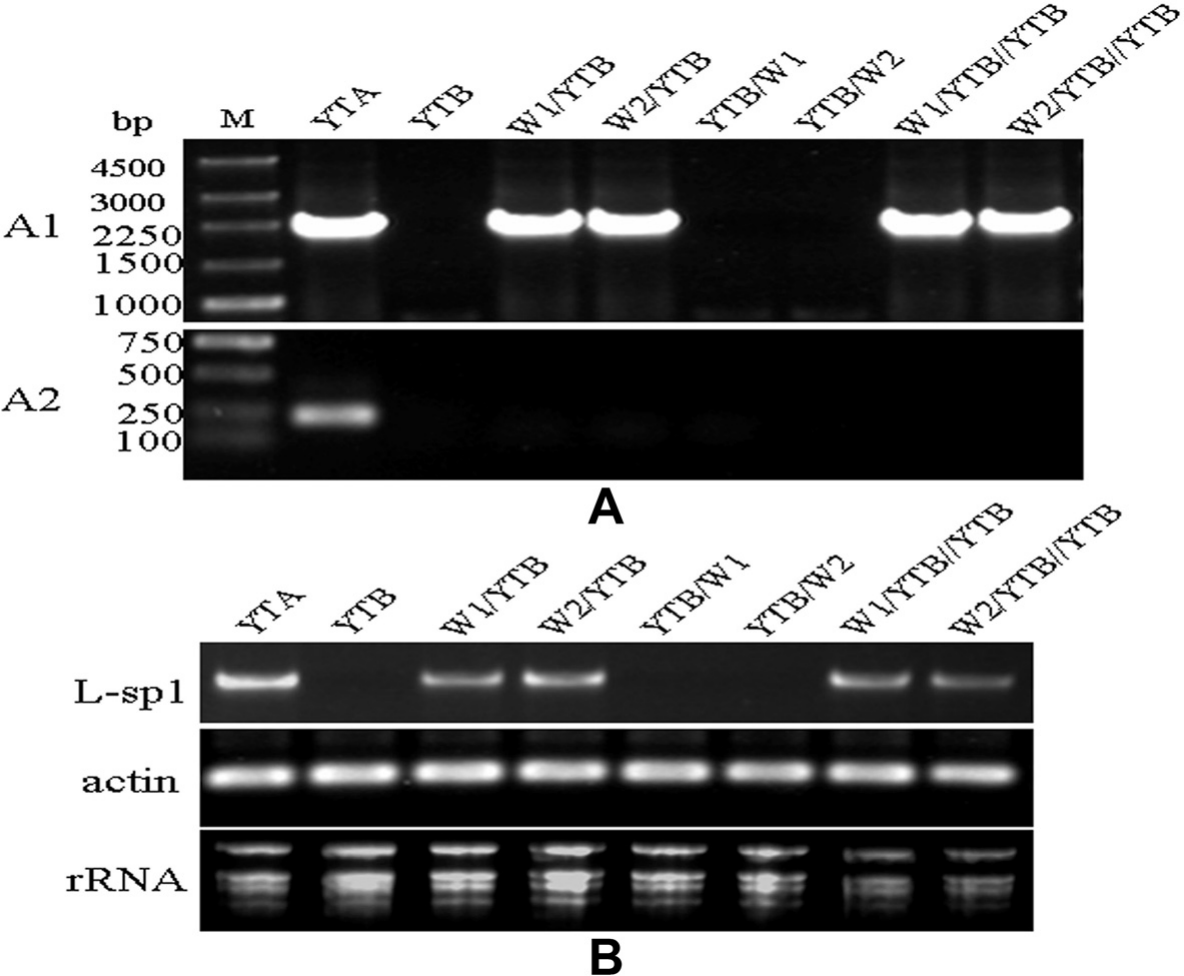
Genetic and transcript analysis of L-sp1 in the newly developed CMS lines. **A**: Genetic analysis: A1, Genetic pattern of L-sp1; A2, Genetic pattern of *orfH79*. **B**: Transcript analysis. M: DNA markers: 250-bp DNA ladder/DL2000. w1: accession 103423; w2: accession 105698.

PCR amplifications were performed using mtDNA from YTA, YTB, w1/YTB, w2/YTB, YTB/w1, YTB/w2, w1/YTB//YTB, and w2/YTB//YTB plants. When using L-sp1 specific primers H1 and H2, an L-sp1 amplicon was observed in plants with the same cytoplasmic background as w1 and w2 (w1/YTB, w2/YTB, w1/YTB//YTB, and w2/YTB//YTB), while the L-sp1 amplicon was not amplified from plants with a different cytoplasm background than w1 and w2 (YTB, YTB/w1, and YTB/w2). This revealed that L-sp1 showed cytoplasmic or maternal inheritance. In addition, the HL-CMS gene *orfH79* was not amplified from the above plants (with the exception of YTA) when using the *orfH79* primer set (O1 and O2) (Figure 6A).

To examine the expression manner of L-sp1 in the F_1_ and backcrossed BC_1_F_1_ w1 and w2 backgrounds, RT-PCR was performed using total RNA from rice anthers with L-sp1 as probe. L-sp1 was found to be expressed in the anthers of w1/YTB, w2/YTB, w1/YTB//YTB, and w2/YTB//YTB in a similar manner to YTA (Figure 6B).

## Discussion

The DNA sequence analysis results suggest no homologous sequence exists between L-sp1 and *orfH79* or *atp6-orfH79*. In wild rice, 19 of 102 investigated wild rice accessions could amplify PCR products of ~240 bp when using *orfH79* sequence-specific primers O1 and O2, a similar product was amplified from YTA. Sequencing of these 19 PCR fragments revealed that eight of the DNA sequences had the same single-nucleotide polymorphism (SNP) as *orf79* in BT-CMS [12], while the remaining 11 accessions had the same SNP as *orfH79* in HL-CMS [33]. In addition, a 2176-bp fragment amplified from L-sp1 specific primers was found present in 11 of 102 wild rice accessions. Nine of these eleven accessions (82042, 101978, 103415, 103835, 105712, 106153, 106321, 100968, and 105661) contain both *orfH79* and L-sp1 sequences, while the other two accessions (103423, 105698) contain only L-sp1. Previous studies documented four completely sterile alloplasmic CMS lines (w15A, w20A, w34A, w46A), developed from w15 (101971), w20 (103836), w34 (105419), and w46 (106321) by successive recurrent backcrossing of sterile plants from a BC_1_F_1_ population with the HL maintainer YTB, respectively [34]. Using the same method, two nearly sterile CMS lines were developed from w1 and w2 accessions carrying L-sp1.

Recent studies revealed CMS-associated mitotypes are compatible with *Rf*-candidate-related nucleotypes, and CMS and *Rf* have a parallel evolutionary relationship in *Oryza* [28]. Several studies suggest that different *Rf* alleles interact with CMS in a gene-for-gene manner. Therefore, various *Rf* loci are determined by the multiple CMS systems existing in the natural populations within plant species [29-32]. We plan to further analyze the restoration and maintenance relationship, and the fertility restoring model of the two CMS lines developed in this study.

## Conclusions

L-sp1 is a 2176-bp CMS-associated chimeric and single-copy DNA fragment found in the mitochondrial genome. It could be expressed in the anthers of all low natural fertility plants or sterile plants with a similar cytoplasm background. Therefore, L-sp1 can be used for both identification of the cytoplasmic background in marker-assisted selection programs and in the development of new CMS lines.

## Methods

### Plant materials

One hundred and two accessions of AA-genome wild rice were obtained from the International Rice Research Institute (IRRI; Los Baños, Laguna, Philippines; Additional file 1: Table S1). These included 22 parent plants, F_1_ hybrids obtained from three types of CMS/*Rf* system (HL-, WA-, and BT-type), and six other CMS lines, each of which had different cytoplasms but identical Shijing B (SJB, a Japonica rice maintainer line) nucleus backgrounds (Table 1). All plant materials were planted in the experimental field within the South-Central University for Nationalities campus in Wuhan, Hubei province in China during summers and in Lingshui, Hainan province in China during winters of 2009 to 2013.

### Isolation of mitochondrial DNA and nuclear DNA

To isolate mtDNA, 20 g of young leaves were harvested using the modified method reported by Yi et al. [33], following etiolation they were homogenized in 80 mL of homogenizing buffer (pH7.2) containing 0.4 M mannitol, 40 mM MOPS, 1 mM EDTA, 0.05% cysteine, 0.1% BSA and 0.03% mercaptoethonal. After differential centrifugation and DNase I processing (Promega, USA), the pellet was resuspended with lysis buffer and fixed at room temperature for 5 min. Following phenol-chloroform extraction, DNA was precipitated with ethanol.

Total nuclear genomic DNA was isolated from green leaves using the modified method described by Zhang et al. [35]. DNA quality and quantity were estimated spectrophotometrically using a specific amount of lambda DNA (MBI, USA) on an agarose gel, and by visualizing under ultraviolet light.

### Polymerase chain reaction amplification

DNA amplification was performed in a programmable thermal controller (PTC-100, MJ Research, USA) using the following program: 5 min at 94°C; 35 cycles of 1 min at 94°C, 1 min at 38°C (RAPD analysis) or 58°C (SCAR analysis), 1.5 min at 72°C; 8-min final extension at 72°C. PCRs were performed in a 25-μL reaction volume (10 mM Tris–HCl (pH 8.8), 25 mM KCl, 1.5 mM MgCl_2_, 0.8 mM dNTPs, 0.2 mM random primer (10 nucleotides), 100 ng genomic DNA, and 1 unit of Taq polymerase (Takara, Japan). Amplified RAPD fragments were electrophoretically separated using 1.5% agarose gels, stained with ethidium bromide, and photographed under ultraviolet light using Gel Doc2000 (Bio-Rad, USA). Molecular weights were estimated using a molecular marker (DL2000, Takara).

### DNA sequencing and SCAR development

DNA polymorphism bands of RAPD markers were isolated and collected from agarose gels using a PCR purification kit according to manufacturer’s specifications (MBI). Purified PCR products were cloned using a pGEM-T easy system I kit (Promega, USA) and sequenced by Shanghai Sangon Biological Engineering Technology and Services (Shanghai, China). The polymorphism sequences were used to design new primers and develop SCAR markers linked to different CMS types. The newly developed primers were used to amplify mitochondrial DNA from all materials mentioned in this study.

### Southern hybridization

L-sp1 (2176 bp) and N-atp6 probes were amplified using primers based on corresponding mitochondrial genomic sequences of rice. Mitochondrial DNA (20 μg) was separated on 0.8% agarose gels following digestion with either *Eco*RI or *Bam*HI (New England Biolabs, USA) and transferred to Hybond N^+^-nylon membranes. Probes were radioactively labeled by random priming with α-^32^P-dCTP. Southern hybridization was performed in hybridization buffer at 65°C for 16 h. The membrane was washed twice at room temperature for 15 min with 2 × SSC containing 0.1% sodium dodecyl sulfate (SDS) and at 60°C for 30 min with 0.1 × SSC containing 0.1% SDS, and was then autoradiographed.

### Transcript analysis

Total RNA was isolated from rice anthers using TRIzol reagent according to the manufacturer’s instructions (Invitrogen, USA). It was then extracted with chloroform, precipitated in isopropyl alcohol, and rinsed with ethanol before being dissolved in RNase-free water. RNase-Free DNase I (Promega, USA)was added to remove any possible genomic DNA contaminants. Synthesis of first-strand cDNA was obtained from the total RNA using a cDNA Synthesis Kit (Toyobo, Japan). The RT-PCR reaction was terminated after 22 cycles, and rice *actin* was used as a control. All assays were repeated at least three times.

## Additional file

Additional file 1: Table S1. All wild rice in the study.

## Abbreviations

CMS: Cytoplasmic male sterility
ORFs: Open reading frames
BLAST: Basic Local Alignment Search Tool
YTA: Yuetai A
YTB: Yuetai B
*Rf*: Fertility restorer
WA: Wild-abortive
HL: Honglian
BT: Boro II
SCAR: Sequence-characterized amplified region
RAPD: Random amplification of polymorphic DNA
MAS: Marker-assisted selection
IRRI: International Rice Research Institute
SJB: Shijing B
bp: Base pairs
DNase: Deoxyribonuclease
RNase: Ribonuclease
dNTP: Deoxynucleotide-5/-triphosphate
EDTA: Ethylenediamine tetraacetic acid
MOPS: 3- (N-Morpholino) propanesulfonic acid
BSA: Bovine Serum Albumin
PCR: Polymerase chain reaction
