# Supplementary material for: Molecular characterization and application of a novel cytoplasmic male sterility-associated mitochondrial sequence in rice
Source: BMC Genet. 2015 Apr 30;16:45. doi: 10.1186/s12863-015-0205-0 (PMC4415283; doi:10.1186/s12863-015-0205-0)
Supplement: Additional file 1: Table S1. — All wild rice in the study. [file 12863_2015_205_MOESM1_ESM.doc]

Supplementary table 1 All wild rice in the study

| No. | Species name | Accession | Origin | No. | Species name | Accession | Origin |
| --- | --- | --- | --- | --- | --- | --- | --- |
| 1 | *O.nivara* | 103835 | Bangladesh | 37 | *O.rufipogon* | 105349 | India |
| 2 | *O.nivara* | 103841 | Bangladesh | 38 | *O.rufipogon* | 105079 | India |
| 3 | *O.nivara* | 103836 | Bangladesh | 39 | *O.rufipogon* | 106083 | India |
| 4 | *O.nivara* | 105712 | Cambodia | 40 | *O.rufipogon* | 106158 | Laos |
| 5 | *O.nivara* | 105728 | Cambodia | 41 | *O.rufipogon* | 106145 | Laos |
| 6 | *O.nivara* | 106309 | Cambodia | 42 | *O.rufipogon* | 106161 | Laos |
| 7 | *O.nivara* | 103821 | China | 43 | *O.rufipogon* | 105491 | Malaysia |
| 8 | *O.nivara* | 103824 | China | 44 | *O.rufipogon* | 106036 | Malaysia |
| 9 | *O.nivara* | 81857 | India | 45 | *O.rufipogon* | 106386 | Myanmar |
| 10 | *O.nivara* | 101978 | India | 46 | *O.rufipogon* | 105698 | Nepal |
| 11 | *O.nivara* | 102163 | India | 47 | *O.rufipogon* | 105696 | Nepal |
| 12 | *O.nivara* | 102167 | India | 48 | *O.rufipogon* | 106268 | New Guinea |
| 13 | *O.nivara* | 101971 | India | 49 | *O.rufipogon* | 106281 | New Guinea |
| 14 | *O.nivara* | 104705 | India | 50 | *O.rufipogon* | 101075 | Philippines |
| 15 | *O.nivara* | 106148 | Laos | 51 | *O.rufipogon* | 103305 | Philippines |
| 16 | *O.nivara* | 106153 | Laos | 52 | *O.rufipogon* | 103423 | Sri Lanka |
| 17 | *O.nivara* | 106344 | Myanmar | 53 | *O.rufipogon* | 104599 | Sri Lanka |
| 18 | *O.nivara* | 106345 | Myanmar | 54 | *O.rufipogon* | 100219 | Thailand |
| 19 | *O.nivara* | 105704 | Nepal | 55 | *O.rufipogon* | 101941 | Thailand |
| 20 | *O.nivara* | 105706 | Nepal | 56 | *O.rufipogon* | 101941 | Vietnam |
| 21 | *O.nivara* | 103415 | Sri Lanka | 57 | *O.rufipogon* | 106516 | Vietnam |
| 22 | *O.nivara* | 103419 | Sri Lanka | 58 | *O.glaberrima* | 101855 | Burkina Faso |
| 23 | *O.nivara* | 104612 | Sri Lanka | 59 | *O.glaberrima* | 103590 | Cameroon |
| 24 | *O.nivara* | 104650 | Thailand | 60 | *O.glaberrima* | 104206 | Ghana |
| 25 | *O.nivara* | 104659 | Thailand | 61 | *O.glaberrima* | 102641 | Liberia |
| 26 | *O.rufipogon* | 105887 | Bangladesh | 62 | *O.glaberrima* | 104285 | Mali |
| 27 | *O.rufipogon* | 103844 | Bangladesh | 63 | *O.glaberrima* | 104540 | Nigeria |
| 28 | *O.rufipogon* | 105902 | Bangladesh | 64 | *O.glaberrima* | 104545 | Nigeria |
| 29 | *O.rufipogon* | 105735 | Cambodia | 65 | *O.glaberrima* | 104007 | Nigeria |
| 30 | *O.rufipogon* | 106321 | Cambodia | 66 | *O.glaberrima* | 104267 | Nigeria |
| 31 | *O.rufipogon* | 104057 | China | 67 | *O.glaberrima* | 101791 | Senegal |
| 32 | *O.rufipogon* | 105400 | China | 68 | *O.barthii* | 101255 | Cameroon |
| 33 | *O.rufipogon* | Wdx-2 | China | 69 | *O.barthii* | 104061 | Cameroon |
| 34 | *O.rufipogon* | 101193 | China | 70 | *O.barthii* | 104131 | Cameroon |
| 35 | *O.rufipogon* | 101974 | India | 71 | *O.barthii* | 104136 | Cameroon |
| 36 | *O.rufipogon* | 104709 | India | 72 | *O.barthii* | 106149 | Guinea |
| 73 | *O.barthii* | 103534 | Mali | 88 | *O.longistaminata* | 103890 | Senegal |
| 74 | *O.barthii* | 104078 | Nigeria | 89 | *O.longistaminata* | 103905 | Tanzania |
| 75 | *O.barthii* | 105609 | Nigeria | 90 | *O.longistaminata* | 81965 | Zambia |
| 76 | *O.barthii* | 103909 | Tanzania | 91 | *O.longistaminata* | 81951 | Zambia |
| 77 | *O.glumaepatula* | 105661 | Brazil | 92 | *O.meridionalis* | 104085 | Australia |
| 78 | *O.glumaepatula* | 105668 | Brazil | 93 | *O.meridionalis* | 105281 | Australia |
| 79 | *O.glumaepatula* | 106233 | Brazil | 94 | *O.meridionalis* | 105289 | Australia |
| 80 | *O.glumaepatula* | 105662 | Brazil | 95 | *O.meridionalis* | 105229 | Australia |
| 81 | *O.glumaepatula* | 105561 | Colombia | 96 | *O.meridionalis* | 105301 | Australia |
| 82 | *O.glumaepatula* | 100184 | Cuba | 97 | *O.meridionalis* | 105306 | Australia |
| 83 | *O.glumaepatula* | 100968 | Suriname | 98 | *O.meridionalis* | 82041 | Australia |
| 84 | *O.longistaminata* | 105198 | Ethiopia | 99 | *O.meridionalis* | 82042 | Australia |
| 85 | *O.longistaminata* | 105206 | Ethiopia | 100 | *O.meridionalis* | 101411 | Australia |
| 86 | *O.longistaminata* | 101213 | Ivory Coast | 101 | *O.meridionalis* | 105283 | Australia |
| 87 | *O.longistaminata* | 105076 | Nigeria | 102 | *O.meridionalis* | 105303 | Australia |

Note: Accession numbers derived from IRRI.
